# Remarkable response to CDK4/6 inhibitor–based endocrine therapy in HR+/HER2− metastatic male breast cancer with visceral crisis: a case report

**DOI:** 10.3389/fonc.2025.1753700

**Published:** 2026-01-02

**Authors:** Jianfeng Liu, Wei Chen, Jian Wang, Xianchen Wang

**Affiliations:** Galactophore Healthcare Department, The Affiliated Weihai Second Municipal Hospital of Qingdao University, Weihai, Shandong, China

**Keywords:** CDK4/6 inhibitor, chemotherapy resistance, HR+/HER2-, male breast cancer, visceral crisis

## Abstract

Male breast cancer (MBC) is rare, and visceral crisis is an exceptionally uncommon yet life-threatening presentation in this population. Although current guidelines recommend combination chemotherapy as first-line therapy for visceral crisis, responses are often inadequate, and evidence for alternative approaches in men is scarce. We report a case of HR+/HER2- metastatic MBC complicated by visceral crisis. Despite first-line chemotherapy, the patient showed swift clinical worsening accompanied by radiographic progression. In view of the lack of treatment response, a CDK4/6 inhibitor–based endocrine regimen was initiated, achieving substantial tumor regression and prolonged disease control. This case suggests that CDK4/6 inhibitor–based endocrine therapy can induce meaningful disease reversal even in visceral crisis and may be a feasible option for selected HR+/HER2− MBC patients after chemotherapy failure. Our case suggests that CDK4/6 inhibitor–based endocrine therapy may offer clinical benefit even in the setting of visceral crisis after chemotherapy failure, indicating that treatment sequencing in such scenarios may merit further consideration.

## Introduction

Male breast cancer (MBC) is a rare malignancy accounting for less than 1% of all breast cancer cases, and its incidence has been gradually increasing, particularly among older men (1, 2). Because of low awareness and delayed diagnosis, many patients present with advanced disease, with a notable proportion diagnosed at stage III–IV (3). MBC typically occurs later in life, with a mean diagnostic age above 60 years across multiple populations (4, 5). Histologically, invasive ductal carcinoma is the predominant subtype, and most tumors are hormone receptor–positive, with estrogen and progesterone receptor expression exceeding 90% in most series (6–8).

Visceral crisis represents a life-threatening manifestation of metastatic breast cancer and is characterized by severe organ dysfunction, which is assessed based on clinical signs, symptoms, laboratory abnormalities, and rapid disease progression (9). Although visceral crisis occurs most frequently in HR-positive/HER2-negative, reports involving male patients are exceedingly rare (10). Current guidelines recommend combination chemotherapy as the preferred initial treatment for visceral crisis, aiming to achieve prompt disease control; however, outcomes remain suboptimal (11). Evidence supporting CDK4/6 inhibitor–based endocrine therapy in the setting of visceral crisis is sparse, and optimal sequencing in male patients is undefined. Here, we describe a rare case of HR+/HER2− metastatic MBC complicated by visceral crisis, in which the patient demonstrated rapid deterioration during first-line chemotherapy but achieved a remarkable and durable response after switching to a CDK4/6 inhibitor–based endocrine regimen. This case highlights the potential value of personalized therapeutic strategies in male breast cancer, extending beyond conventional guideline-recommended approaches.

## Case presentation

A 61-year-old man presented to our hospital in April 9, 2024 for evaluation of a left breast mass that had been incidentally noted two years earlier. The mass was initially peanut-sized and later developed mild surface erythema and scabbing following spontaneous ulceration, but remained painless and stable in size. The patient reported no fever, chills, weight loss, or nipple discharge, and had no personal or family history of breast cancer or related malignancies.

**Figure 1:**
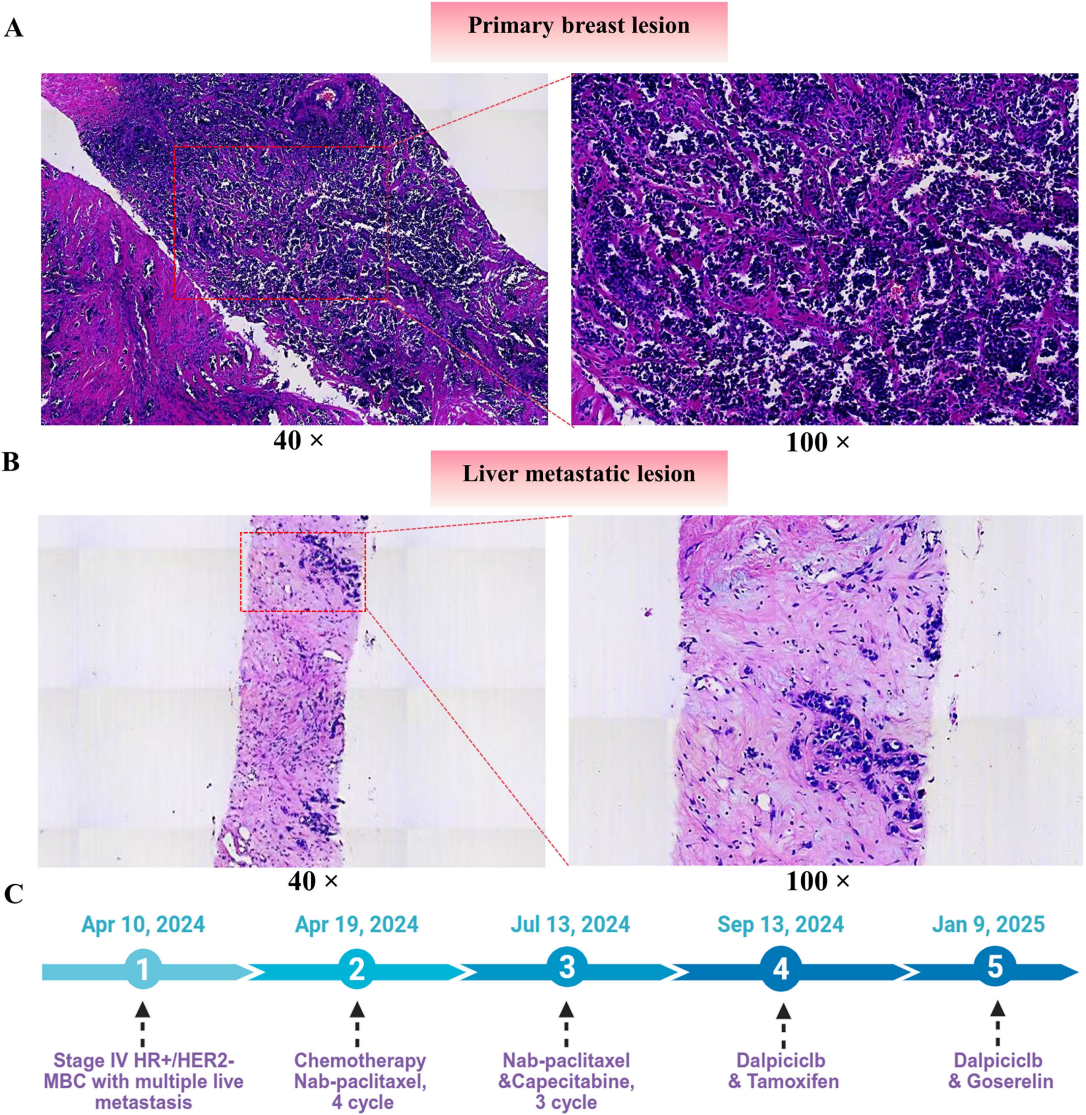
Histopathology of the primary breast tumor and liver metastasis and treatment timeline. **(A)** Representative hematoxylin and eosin (H&E) staining of the primary breast lesion at low (40×, left) and higher (100×, right) magnification. **(B)** Representative H&E staining of the liver metastatic lesion at low (40×, left) and higher (100×, right) magnification. **(C)** Clinical course and treatment timeline.

Physical examination revealed a firm, irregular 2.0 × 1.5 cm mass beneath the left nipple with overlying erythema and scabbing, but no tenderness or discharge. No axillary lymphadenopathy was detected. Breast ultrasound showed a 29 × 28 × 25 mm hypoechoic mass with irregular margins and internal vascularity (BI-RADS 4C). Mammography demonstrated a high-density lobulated mass with microcalcifications. Abdominal MRI revealed multiple liver lesions, the largest measuring 68 × 45 × 70 mm, suggestive of metastases, along with enlarged lymph nodes in the porta hepatis and retroperitoneum. Bone scintigraphy indicated multifocal skeletal metastases. On April 10, 2024, ultrasound-guided core needle biopsies of the left breast mass and the enlarged left supraclavicular lymph node were performed under local anesthesia. Histopathological examination of the breast specimen revealed high-grade invasive ductal carcinoma characterized by marked nuclear atypia and frequent mitoses (**Figure 1A**). Immunohistochemistry (IHC) showed ER 90% (2+), PR 90% (3+), AR 70% (2+), HER2 (1+), and Ki-67 50%. Supraclavicular lymph node and liver biopsies confirmed metastatic carcinoma with concordant IHC findings (**Figure 1B**). HER2 FISH was negative. In addition, the patient presented with extensive liver metastases accompanied by abnormal liver function. According to the 5th ESO–ESMO International Consensus Guidelines for Advanced Breast Cancer (12), this condition meets the diagnostic criteria for liver crisis. Finally, the disease was classified as *de novo* stage IV HR+/HER2− breast cancer with liver and bone metastases.

**Figure 2:**
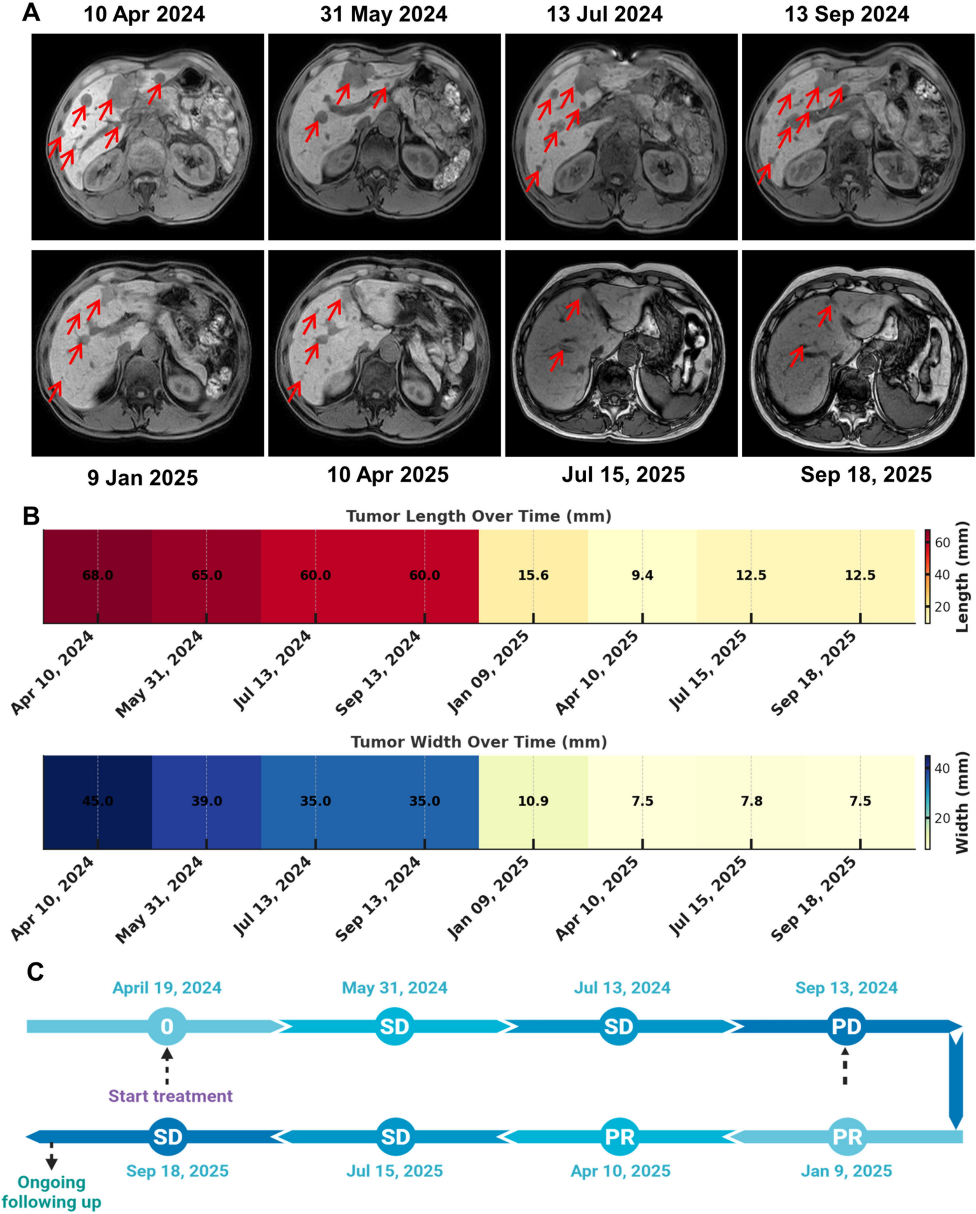
Longitudinal radiographic assessment of liver metastases and clinical response. **(A)** Representative axial abdominal MRI images acquired at serial time points. Red arrows indicate hepatic metastatic lesions. **(B)** Heatmaps showing changes in target-lesion size over time, including maximal tumor length (top) and width (bottom). **(C)** Schematic timeline of systemic therapy and best overall response according to radiographic evaluation, indicating stable disease (SD), progressive disease (PD), and partial response (PR) across follow-up.

Given the extensive liver metastases, the patient was started on nab-paclitaxel as salvage chemotherapy (200 mg on Days 1 and 8 of a 21-day cycle) beginning on April 19, 2024. Zoledronic acid was administered concurrently to prevent skeletal-related events. Following chemotherapy, the patient developed bone marrow suppression, which was managed with G-CSF support (**Figure 1C**). Target hepatic lesions were selected and measured according to RECIST v1.1. After two cycles, MRI on May 31, 2024, showed slight shrinkage of the hepatic lesions, and the treatment response was classified as stable disease (SD) according to RECIST criteria. After four cycles, MRI on July 13, 2024, demonstrated only minimal additional reduction, with the response remaining SD, which prompted the initiation of capecitabine (1 g twice daily on Days 1–14 of a 21-day cycle). After seven cycles, MRI on September 13, 2024, demonstrated a mixed hepatic response with enlargement of certain lesions. PET-CT further confirmed metabolically active liver metastases (SUVmax 8.4), along with multifocal osteoblastic lesions and mildly FDG-avid left axillary lymph nodes, indicating persistent residual disease and meeting the criteria for progressive disease (PD) (**Figure 2A**).

However, as the treatment progressed, chemotherapy became less effective. Based on clinical guidelines, the regimen was switched to endocrine therapy combined with CDK4/6 inhibition. The patient began dalpiciclib (150 mg daily on Days 1–21 of a 28-day cycle) together with androgen deprivation therapy using goserelin (3.6 mg every 28 days) and letrozole (2.5 mg daily). Zoledronic acid was continued. After three months, MRI on January 9, 2025, showed marked shrinkage of the liver metastases, consistent with a partial response (PR) (**Figure 2B**). However, the patient reported severe hot flashes following the administration of goserelin and requested discontinuation of the depot formulation. Consequently, goserelin was temporarily withheld, and letrozole was replaced with tamoxifen (10 mg twice daily), while dalpiciclib was continued. In addition, the patient developed Grade II bone marrow suppression, presenting with leukopenia and neutropenia, which improved significantly after treatment with the oral leukocyte-stimulating agent levamisole. MRI on April 10, 2025, demonstrated further reduction in hepatic lesions, sustaining the PR. The patient later agreed to resume medical castration. At the most recent follow-up on September 18, 2025, MRI showed stable treated liver metastases (SD) (**Figure 2C**). Total bilirubin returned to the normal range, while ALT, AST, and GGT slightly increased, and alkaline phosphatase remained normal. Currently, this patient maintained good overall condition, was able to perform light physical activities, and reported normal appetite, sleep, and bowel and bladder function, with no noticeable weight changes. This patient remains on dalpiciclib, tamoxifen, and zoledronic acid.

## Discussion

The optimal management of visceral crisis in MBC remains uncertain, as current recommendations are largely extrapolated from female breast cancer populations. Although NCCN and ESMO guidelines prioritize combination chemotherapy for its presumed rapid onset, real-world outcomes are often suboptimal in patients with substantial hepatic tumor burden or declining liver function, and these limitations are further magnified in visceral crisis, where rapid organ recovery is critical (13). Our patient, who presented with *de novo* stage IV HR+/HER2− MBC with extensive liver and bone metastases, received guideline-concordant chemotherapy with nab-paclitaxel followed by capecitabine, yet ultimately showed disease progression with mixed radiologic responses and persistent metabolic activity on PET-CT. This clinical course underscores a key challenge in visceral crisis: cytotoxic therapy may be insufficient for patients with heavy disease burden, emerging chemoresistance, or compromised organ function, and treatment-related toxicity such as bone marrow suppression can further hinder care. Collectively, this case highlights a real-world dilemma in visceral crisis management and reinforces the need to explore therapeutic strategies beyond guideline-directed chemotherapy.

Cell-cycle progression through the G1/S checkpoint is governed by the cyclin D–CDK4/6–Rb signaling axis (14). Activation of CDK4/6 leads to Rb phosphorylation, release of E2F transcription factors, and S-phase entry. CDK4/6 inhibitors block Rb phosphorylation, induce G1 arrest, and suppress tumor proliferation, forming the mechanistic rationale for their use in HR+/HER2− breast cancer (15). Palbociclib, ribociclib, and abemaciclib have become standard-of-care therapies for women with advanced HR+/HER2− disease, significantly prolonging progression-free and overall survival (16–18). Although MBC displays similar hormone dependence, evidence supporting CDK4/6 inhibitor use in men remains limited. Real-world analyses, such as the CompLEEment-1 study, suggest that ribociclib combined with letrozole is effective and well tolerated in male patients, with lower rates of grade ≥3 neutropenia compared to the overall study population (41.0% vs. 57.2%) (19, 20). Conversely, isolated cases have described primary resistance to CDK4/6 inhibitors with subsequent responses to tamoxifen, pointing toward potential sex-specific biological differences and reinforcing the importance of individualized therapy (21). Additionally, emerging reports of toxicities—such as vitiligo-like depigmentation and neurocognitive impairment (22–24)—highlight the need for careful monitoring and patient selection. Although CDK4/6 inhibitors remain the cornerstone of treatment for HR+/HER2− metastatic breast cancer, emerging evidence suggests that some patients may derive greater benefit from chemotherapy as initial therapy. For example, a recent multicenter real-world study comparing first-line chemotherapy with CDK4/6 inhibitors in HR-positive, HER2-negative breast cancer with liver metastases demonstrated that, although progression-free survival favored CDK4/6 inhibitor therapy (10.9 vs. 4.8 months), overall survival was significantly longer with chemotherapy (42.2 vs. 25.9 months) (25).

Against this background, switching to CDK4/6 inhibitor–based endocrine therapy in our patient represented a biologically driven shift after chemotherapy failure. CDK4/6 inhibitors are known to exert early antiproliferative effects, which may be particularly advantageous in high–tumor-burden settings such as liver crisis. Although men remain underrepresented in clinical trials, retrospective data indicate similar therapeutic responses between sexes. Consistent with this, our patient experienced marked radiologic tumor regression within three months of initiating dalpiciclib combined with androgen deprivation therapy and aromatase inhibition, followed by sustained disease control. Importantly, several hepatic lesions shrank significantly, reversing the progression of liver crisis and enabling long-term clinical stabilization.

The endocrine component of treatment in male patients warrants additional consideration. Unlike women, men require medical castration to suppress testicular androgen production, as aromatase inhibitors alone do not adequately reduce circulating androgen levels. In this case, goserelin-induced severe hot flashes led to temporary discontinuation of androgen deprivation therapy, necessitating a switch from letrozole to tamoxifen. Despite these changes, the patient maintained a favorable response, suggesting that sustained CDK4/6 inhibition may be a key driver of disease control, while the endocrine backbone can be individualized based on tolerability. This adaptability underscores the need for tailored treatment strategies in MBC, where physiologic differences and toxicity profiles may differ from those of female patients.

The clinical implications of this case are noteworthy. It suggests that CDK4/6 inhibitor–based endocrine therapy may serve as an effective alternative for selected HR+/HER2− MBC patients with visceral crisis, particularly after chemotherapy failure. The case also highlights the need to move beyond rigid, guideline-driven chemotherapy in visceral crisis toward a more biology-informed treatment approach, and emphasizes the importance of sex-inclusive research in male breast cancer. However, the findings should be interpreted with caution due to the inherent limitations of a single case, including factors such as tumor biology, hormone receptor expression, performance status, and treatment adherence. Additionally, the lack of male-specific data in visceral crisis further limits the generalizability of these results. Larger, more diverse clinical studies are needed to better define the role of CDK4/6 inhibitors in visceral crisis, particularly in male patients.

In conclusion, our case suggests that CDK4/6 inhibitor–based endocrine therapy may offer rapid and durable disease control even in the setting of visceral crisis after chemotherapy failure. While these observations are hypothesis-generating, they indicate that CDK4/6 inhibition could be considered a potential therapeutic option for carefully selected HR+/HER2− MBC patients experiencing visceral crisis. Further research is warranted to better define its role and identify patients most likely to benefit.

## Data Availability

The original contributions presented in the study are included in the article/Supplementary Material. Further inquiries can be directed to the corresponding authors.
